# 肺癌早期诊断研究进展

**DOI:** 10.3779/j.issn.1009-3419.2011.05.09

**Published:** 2011-05-20

**Affiliations:** 100021 北京，中国医学科学院北京协和医学院，肿瘤医院肿瘤研究所，分子肿瘤学国家重点实验室 State Key Laboratory of Molecular Oncology, Cancer Institute (Hospital), Peking Union Medical College and Chinese Academy of Medical Sciences, Beijing 100021, China

**Keywords:** 肺肿瘤, 早期诊断, 分子标志物, Lung neoplasms, Early diagnosis, Biomarkers

## Abstract

肺癌是绝大多数国家主要的癌症死亡原因，早期诊断和早期治疗尤为重要。以胸部X光片、螺旋CT、支气管内镜、痰液细胞学等检测手段用于肺癌的筛检和早诊已有较多报道，但鉴于以上检查手段的敏感性、特异性、适用度等方面的局限，近年来国内外学者对有关肺癌早期诊断的分子标志物做了大量有益的探索，本文拟就该领域的相关进展作一综述。

## Abstract (en)

Lung cancer is the most common cause of cancer death worldwide. Early diagnosis and early treatment are of great importance. Chest X-ray, spinal CT, bronchoscopy and sputum cytology have long been used as screening or diagnostic modalities for early detection of lung cancer. However, these methods have limitations in sensitivity, specificity or utility in some degree. In recent years, researchers all over the world have done lots of work on finding and identifying biomarkers for the early diagnosis of lung cancer. In this paper, recent deveploments in this field are reviewed.

肺癌是当今世界各国最常见的恶性肿瘤，其死亡率居于各种肿瘤的首位，对人类健康和生命构成极大威胁。在我国，肺癌每年约致40万例患者死亡^[1]^。然而，研究显示Ⅰ期肺癌术后10年生存率可达到92%^[2]^。因此，降低肺癌患者死亡率的关键在于早期诊断和早期治疗。

关于早期肺癌的概念在国际上依然存在着不同的观点，有学者认为原位癌和Ⅰa期肺癌为早期肺癌^[3]^，也有认为早期肺癌是指全部Ⅰ期肺癌^[4]^，还有学者认为Ⅰ期和Ⅱ期都属于早期^[5]^，另有认为直径 < 3 cm即为早期肺癌^[6]^。随着肺癌病因学、分子标志物研究工作的进展，以及低剂量螺旋CT、荧光纤维支气管镜和液基细胞学在临床的应用，肺癌的早期诊断取得了长足的进步，本文拟就该领域的研究进展作一综述。

## 1 肺癌早期诊断方法

### 1.1 影像学检查

胸部X光片优点是能观察胸部各种结构的全貌，经济且方便。但因其分辨率低、组织结构互相重叠，在肺癌早期诊断方面并不具优势，更精确的数字化成像可能是未来的发展方向之一^[7]^。低剂量螺旋CT（low-dose CT, LDCT）优点是可以检测到2 mm-3 mm的小结节，尽管有报道LDCT筛查不能明显提高生存率^[8]^，但在全美范围内进行的大型随机肺癌筛查（National Lung Screening Trial, NLST）研究的初步结果显示，其可降低肺癌死亡率达20%^[9]^。鉴于现有研究数据不一致，2010年肺癌NCCN指南暂不推荐在非小细胞肺癌临床实践中常规进行CT筛查。PET虽在肺癌早期诊断应用方面并无定论，但其已成为辅助肺癌分期的重要手段，采用PET/CT对早期患者进行准确分期，可以避免不恰当的手术^[10]^。

### 1.2 支气管内镜

各种支气管内镜的发展使得其在肺癌早期诊断中发挥越来越重要的作用。自发荧光支气管镜（autofluorecence bronchoscopy, AFB）较之常规白光支气管镜（white-light bronchoscop, WLB）不仅可提高早期癌变的检出率，也可更清晰地显示肿瘤的轮廓。一项在大于1, 000例的比较研究^[11]^中显示，单用WLB可检测到40%的侵袭前病例，而联用AFB可将检出率提高到80%。但AFB对于侵袭前病变诊断特异性较低，自发荧光成像电子支气管镜系统（auto-fluorescence imaging, AFI）可通过检测区域的颜色区分癌前病变和支气管的良性改变，从而解决了这一难题。此外，窄带成像支气管镜（narrow band imaging, NBI）可对支气管粘膜及血管进行清晰显示，故在鳞癌的癌前病变诊断方面具有很大优势。将自发荧光和窄带成像统一结合于电子支气管镜成像系统上可能是未来的发展方向，其可减少临床操作时间，同时可避免一些不必要的活检操作^[12]^。

## 2 肺癌分子标志物

肺癌的发生是多因素致病、多基因参与和多阶段发展的复杂过程，鉴于以上种种检查手段的敏感性、特异性、适用度等方面的局限，近年来国内外学者对有关肺癌早期诊断的分子标志物做了大量有益的探索。

### 2.1 染色体水平

等位基因丢失或杂合性丢失在肺癌早期常被探测到，如3p14、9p21、17p13、13q14、5q21、1q、2q和8q，它们也可在吸烟者或既往吸烟者的组织学正常或增生上皮中探测到。最常发生异常改变的是3p^[13, 14]^，这也是诸多抑癌基因（tumor suppressor gene, TSG）所在的染色体区段。最近一项在痰液中检测染色体非整倍性的病例对照研究^[15]^显示，一组FISH探针（EGFR、MYCC、5p15及CEP6）联合痰细胞学，可提前于常规诊断18个月预测肺癌发生，诊断敏感度和特异性分别为76%和88%。

### 2.2 DNA水平

肿瘤是一种由多个关键基因的遗传学和表观遗传学改变导致的转化细胞克隆增殖的疾病，关键基因的改变可以是胚系突变或体细胞突变。

华盛顿大学基因研究所在188例肺腺癌中对623个与肺癌发生有已知或潜在关系的基因进行测序，发现了多于1, 000个体细胞突变，最终确定了26个最常见的突变，涉及*TP53*、*KRAS*、*STK11*、*EGFR*、*LRP1B*等基因^[16]^。对这些基因进行大样本的临床验证，可能发现有助于早期诊断的标志物。

Belinsky等^[17]^发现一组基因的甲基化在诊断肺癌方面具有较高的敏感度和特异性。他们收取了3, 259例患者的痰液，其中182例被诊断为肺癌，具有高质量DNA的病例有98例，非癌组92例作为对照。共检测了14个基因的甲基化状态，发现6个（*P16*、*MGMT*、*DAPK*、*RASSFIA*、*PAX5β*及*GATA5*）与肺癌发生风险增高相关，其中3个或更多个基因的共同甲基化可增加6.5倍肺癌发生的风险，对肺癌检出的敏感性和特异性均为64%。研究者认为此敏感性还不足以在大规模人群中开展前瞻性研究，有待对候选基因进行更多的验证。

Spira等^[18]^检测了129例接受了支气管镜刷检的吸烟者的支气管上皮细胞，并一直随访到检查者最终获得活检/手术病理结果，有60例被诊断为肺癌，69例无肺癌。研究者用其中77例作为实验组通过表达谱芯片得到一组含80个基因的标志物，并在其余52例样本中进行验证，发现其诊断敏感度和特异性分别为80%和84%。在92例支气管镜未确诊患者中，诊断敏感度和特异性分别为89%和83%。将标志物与支气管镜合用，诊断敏感性和阴性预测率均高达95%，尤其对于Ⅰ期肺癌，标志物诊断的敏感性为90%，而常规支气管镜的敏感性为35%。通过此项研究，Spira等认为在细胞学正常的支气管上皮标本中的基因表达也可作为肺癌的诊断标志物。

关于循环肿瘤细胞DNA的研究^[19, 20]^也有很多，但由于缺乏统一标准、各项研究所采用的界值不一致、可重复性较差等问题，迄今还没有实现临床应用。

### 2.3 蛋白水平

#### 2.3.1 血清蛋白改变

血清蛋白标志物检测具有高效、高灵敏性、性价比高、方便简捷、快速、标本易获取及创伤小的特点。目前在肺癌中研究的血清肿瘤相关抗原标志物有CEA、NSE、TPA、Chromogranine、CA125、CA19-9、Cyfra21-1，敏感性和特异性在50%-90%不等，大部分标志物随病变进展阳性检出率增高。

Garaci等^[21]^在534例个体的外周血中对TLP进行检测，发现其在非小细胞肺癌患者中的检出率为53.1%，而在其它类型肿瘤患者中未检测到。在Ⅰ期肺癌中有75%可检测到TLP。Tarro等^[22]^总结文献报道的肺癌血清标志物研究的数据发现，作为Ⅰ期肺癌诊断标志物，TLP的检出敏感性和特异性分别为66.7%和80%，而其它常见血清标志物CA19-9为33.3%和100%，Cyfra21-1为11.1%和100%，CA125为11.1和100%，CEA为0和100%。

Yang等^[23]^用质谱技术检测了158例肺癌患者和50例对照的血清，其中47例肺癌患者血清与20例正常对照作为实验组，发现5个标志峰，然后在84例肺癌患者和30例正常对照中进行盲性验证，发现检测敏感性为87%，特异性为80%。

Nisman等^[24]^检测了37例肺部良性病变、88例非小细胞肺癌和37例小细胞肺癌患者血清中ProGRP、NSE、CYFRA21-1及CEA的水平，结果发现ProGRP在区分小细胞肺癌和肺部良性病变及非小细胞肺癌方面优于NSE，特异性可达90%以上，ProGRP对于小细胞肺癌的检出敏感性为95%，可能作为小细胞肺癌诊断的一个候选标志物。

肿瘤相关自身抗体的检测近年也有很大发展，一项在125例非小细胞肺癌患者和125例性别、年龄、吸烟状态等因素匹配的正常对照中的研究显示，CFH自身抗体可在51.5%的早期肺癌中检测到，而在对照组中仅有8.0%^[25]^。另有研究显示，一组由6个自身抗体构成的血清蛋白标志在高危人群中诊断肺癌的敏感性和特异性可分别达94.8%和91.1%^[26]^。

#### 2.3.2 组织蛋白（标志）变化

在支气管活检标本中，p53的过表达是一个很有趣的标志物，在化生中是不存在的，但在不典型增生中的存在程度与癌的形成密切相关。一项调查^[27]^结果显示，p53表达阳性率在轻度不典型增生病变中为19%，中度不典型增生为36%，原位癌为59%。一项在378例肺癌手术标本中的免疫组化结果^[28]^显示，UHRF1在几乎一半的肺癌早期病例中均有表达，UHRF1特异表达于癌细胞的细胞核，而在手术切端组织、间质细胞及炎性细胞中均不表达，可能作为肺癌早诊的潜在蛋白标志。

近些年，蛋白质组学的发展以及蛋白芯片的应用使得越来越多的标志物被发现，但还需在组织样本与大规模人群中验证。

### 2.4 RNA水平

Sueoka等^[29]^用Real-time RT-PCR的方法检测了44例肺癌患者、7例肺部增生病变患者、24例肺部良性病变患者及25例正常对照的血浆hnRNPB1水平，发现在肺癌患者中显著高于正常对照及肺部良性病变者（*P* < 0.05）尤其在鳞癌患者中显著高于腺癌患者水平。他们认为其可作为一种无创筛查用于肺癌的早期诊断。Yu等^[30]^在痰液中研究发现一组miRNAs标志（miR-21、miR-486、miR-375和miR-200b）可将肺腺癌患者和正常人群很好的区分开，敏感性和特异性分别达80.6%和91.7%。

还有很多其它mRNA表达谱的研究报道，但缺乏敏感性和特异性数据。

### 2.5 呼出气体

呼出气体是比痰液更易得的标本，在这种样本中寻找肺癌标志物是一项新的有益探索。文献^[31, 32]^报道，呼出气体中一组由VOCs构成的标志物可区分肺癌患者与正常受检者。随着检测技术如TOF MS、PTRMS、SIFT-MS和激光光谱等的不断发展，对呼出气体及呼出气冷凝液中肺癌标志物的研究逐渐深入，但该领域总体仍处于较初级阶段。轻便的检测装置、低廉的检测价格以及结果的易于判读，将会是此方面检测今后应用于临床的重要发展方向^[33]^。

## 3 高危人群易感相关基因

迄今为止，在肺癌中研究者还没有找到一种像APC与家族性结肠息肉病那样关系密切的胚系突变。研究^[34, 35]^认为，遗传有可能独立于吸烟史或环境中烟草暴露剂量而成为发生肺癌的因素。Bailey-Wilson等^[36]^报道6q23-q25可能存在肺癌易感基因。

流行病学研究表明，肺癌易感风险增高与一些遗传性癌症综合征有关，而这往往与*p53*^[37]^、*EGFR*^[38]^等基因的胚系突变有关。大规模全基因组关联性研究^[39, 40]^发现15q24-15q25.1区域的SNP与肺癌发生有关，其中包含两个受尼古丁暴露剂量调控编码烟碱乙酰胆碱受体alpha亚基的基因。另有研究^[41]^显示，位于5p15.33的rs2736100与非吸烟女性患肺腺癌风险显著相关。

Wu等^[42]^在中国人群中发现与肺癌易感风险有显著相关的四个SNP位点，即rs2036534C > T、rs667282C > T、rs12910984G > A和rs6495309T > C，其均位于15q25。该研究组还发现与肺癌发生风险增高显著相关的其它一些位点，如：XRCC1 5'UTR区的SNP-77T > C^[43]^，ERCC1 5’侧翼区的-433T > C和262G > T^[44]^，FEN1启动子区的rs174538G > A和rs4246215G > T^[45]^，MAD1L1和MAD2L1的遗传性变异^[46]^和CASP8启动子区6个核苷酸的缺失多态^[47]^。

表 1综合了近两年肺癌全基因组关联研究（genomewide association study, GWAS）的部分结果。GWAS可同时检测基因组中成百上千的变异，从而发现疾病的候选基因，为人们研究肿瘤易感基因提供了新的方式，对了解肿瘤的发病机制提供了更多的线索。通过GWAS尽管已发现大量与肺癌相关的变异，但仍然仅占整个基因组变异中的一小部分，所起的作用也非常小。而且在这种情况下，很小的一点系统或随机误差都可能导致不正确的结果，故而在GWAS中基因数据的质量控制非常重要^[53]^。再者，正因为GWAS仅关注于每种肿瘤基因组构成中的一小部分基因变异, 低估了肿瘤自然发生的复杂性。因此，对GWAS取得的发现仍需要大量长期的随访方可定论，以阐明关联之下的分子生物学机制，进而评价肺癌的发生风险及疾病转归。

**1:** 肺癌的全基因组关联研究 Genome-wide association study of lung cancer

Study	Region	Location
Landi^[48]^	rs2736100	5p15.33
Yoon^[49]^	rs2131877	3q29
Schwartz^[50]^	rs6587361 and rs181696	chr 1 and chr3q
Kohno^[51]^	HLA-DQA1	6p21.31
Li^[52]^	rs2352028	13q31.3

## 4 问题与展望

肺癌已成为世界癌症死亡的首要原因，人们一直在不断探索肺癌早期诊断的新技术，包括在血清、痰液、呼出气体及支气管活检中寻找用于肺癌早期诊断的标志物，甚至口腔上皮也被用于肺癌检测^[54]^，所发现的大量标志物均需经过多中心大样本的验证。多个标志物的联合应用将会是未来标志物检测的重要发展方向之一。此外，基因工程小鼠模型在肺癌早期诊断研究中得到越来越多的关注，有可能成为今后研究方向之一，多光子显微成像等多种影像技术的发展也使得小鼠模型在肺癌早期诊断中发挥越来越重要的作用^[55]^。

另一方面，在临床中检测特异标志物的仪器设备需要进一步发展。原位癌一般包括4层-38层细胞、且大多数病损有5层细胞的厚度，由非周围型支气管粘膜的异型细胞斑块构成，一般只有几个毫米^[56, 57]^，更何况潜在癌变的细胞克隆远远小于现今测量技术可达的最小阈值，这就可以解释为什么常规手段如胸片、CT和纤维支气管镜在早期诊断方面不那么有效了。需要开发出更准确更敏感的技术，以期对肺癌进行早期诊断。再者，对肿瘤早期的标志物进行动态测定和与影像学方法相结合的分子影像技术的发展，将可能为肺癌的早期发现和早期诊断带来新的希望。有理由相信，随着现代科学技术的发展，21世纪人类对肺癌早诊早治的工作会有更大的作为，将取得更大的成就。
